# Please Read

**Published:** 1877-06-20

**Authors:** 


					﻿Editorial and Miscellaneous.
Please Read.—We send this No. as a
sample to many who are not on our list. We
have this to say to them. We are publishing
a journal which aims to be practical and use-
ful to the busy practitioner. It is not perfect,
is not what we intend to make it, but we be-
lieve, and we have hundreds of testimonials
in proof of the assertion from those who have
been taking it, that for plain and practical in-
formation suited to the wants of the village
and county practioner, it is unequaled by any
journal in the United States. It will cost you
but little to test our opinion in this matter.
We ask you to take our journal for twelve
months and try it. May take it for six
months if you prefer to do so, commencing at
any time, but as your volume would be in-
complete, it is best to take the back numbers
from January.
				

